# Children benefit from gestures to understand degraded speech but to a lesser extent than adults

**DOI:** 10.3389/fpsyg.2023.1305562

**Published:** 2024-01-18

**Authors:** Kazuki Sekine, Aslı Özyürek

**Affiliations:** ^1^Faculty of Human Sciences, Waseda University, Tokorozawa, Japan; ^2^Centre for Language Studies, Radboud University, Nijmegen, Netherlands; ^3^Max Planck Institute for Psycholinguistics, Nijmegen, Netherlands; ^4^Donders Institute for Brain, Cognition and Behaviour, Radboud University, Nijmegen, Netherlands

**Keywords:** co-speech gestures, degraded speech, primary school children, multimodal integration, speech comprehension

## Abstract

The present study investigated to what extent children, compared to adults, benefit from gestures to disambiguate degraded speech by manipulating speech signals and manual modality. Dutch-speaking adults (*N* = 20) and 6- and 7-year-old children (*N* = 15) were presented with a series of video clips in which an actor produced a Dutch action verb with or without an accompanying iconic gesture. Participants were then asked to repeat what they had heard. The speech signal was either clear or altered into 4- or 8-band noise-vocoded speech. Children had more difficulty than adults in disambiguating degraded speech in the speech-only condition. However, when presented with both speech and gestures, children reached a comparable level of accuracy to that of adults in the degraded-speech-only condition. Furthermore, for adults, the enhancement of gestures was greater in the 4-band condition than in the 8-band condition, whereas children showed the opposite pattern. Gestures help children to disambiguate degraded speech, but children need more phonological information than adults to benefit from use of gestures. Children’s multimodal language integration needs to further develop to adapt flexibly to challenging situations such as degraded speech, as tested in our study, or instances where speech is heard with environmental noise or through a face mask.

## 1 Introduction

Children typically communicate with their caregivers in multimodal environments and interact through a variety of modalities, including eye gaze, facial expressions and hand gestures, in addition to speech (e.g., Özçalışkan and Dimitrova, 2013; Çetinçelik et al., 2021). It is known that even young children integrate information from different communicative channels, such as hand gestures and speech. For example, Sekine et al. (2015) showed that children develop the ability to integrate information from gesture and speech from 3 years of age and above (Sekine et al., 2015). This developmental trend has been found by other studies as well (e.g., Stanfield et al., 2014; Glasser et al., 2018). The multimodal nature of interactions may be especially crucial in environments where speech may be degraded, such as in classrooms, sport fields, public transport or when others are wearing masks. Particularly, since the COVID-19 pandemic started, many people interact while wearing masks, which makes speech more unintelligible (Kim and Thompson, 2022).

In these environments where natural language is used, speech is not always clear. We do not know, however, the extent children can integrate multimodal information in such challenging situations. In order to have a full understanding of children’s comprehension skills of multimodal language we need to investigate the flexibility of their system compared to adults. This should be investigated not only in perfect but also in challenging contexts.

Previous studies have shown that gestures, defined as meaningful hand movements that accompany speech, are part of an integrated system of language (McNeill, 1992; Kita and Özyürek, 2003; Özyürek, 2014), both in production and comprehension (Kelly et al., 2010), and both in typical environments and in noisy situations. In line with this integrated view of speech and gesture, it has been also shown that adult listeners are flexible and use gestures to disambiguate comprehending speech with externally- or internally-induced noise, as observed in degraded speech (e.g., Drijvers and Özyürek, 2017, 2020; Schubotz et al., 2020; Wilms et al., 2022), in noisy environments (Kendon, 2004), or in instances involving hearing difficulties (Obermeier et al., 2012). It is not clear, however, whether this flexible integrated system is in place in children as in adults, especially in noisy situations which are more taxing than in clear speech situations. Thus, little is known regarding the extent to which children could benefit from gestures when speech is degraded. Therefore, in the current study, we examined for the first time the enhancement effect of iconic gestures on the comprehension of degraded speech in children, and compared this to adults.

Throughout this paper, we use the term ‘clear speech’ to mean that the speech is not degraded, although we are aware the term is also used in other context for therapeutic techniques utilized with individuals with motor speech disorders and those with hearing loss.

### 1.1 Gesture-speech integration in clear and noisy speech in adults

Speakers use hand gestures to represent iconic semantic information that is relevant to the information conveyed by the concurrent speech. These gestures are referred to as iconic gestures, as they iconically represent concrete aspects of a referent, such as shape, size, motion, or relative position (McNeill, 1992). Such gestures are known to be integrated with linguistic information, accompanying clear speech at the semantic, syntactic, prosodic, discourse and pragmatic levels during both production and comprehension (Kelly et al., 2010; Özyürek, 2014) as well as during the interactive aspects of communication such as in dialog (Rasenberg et al., 2020).

Recent research has shown that such gesture and speech are integrated also in noisy situations. For example, it has been observed that people effectively use and modulate their gestures in adverse listening conditions (Kendon, 2004; Trujillo et al., 2021). Numerous studies have empirically shown that the use of iconic gestures is beneficial for adult listeners when speech is degraded as well (e.g., Rogers, 1978; Holle et al., 2010; Drijvers and Özyürek, 2017; Wilms et al., 2022). For example, Drijvers and Özyürek (2017) examined the enhancement effect of both iconic gestures and lip movements (visible speech) on the comprehension of degraded speech by comparing comprehension among three different noise level conditions: 2-band noise-vocoding (severe noise), 6-band noise-vocoding (moderate noise) and clear speech. Participants were presented with a series of video clips in which an actor recited an action verb with or without a gesture and/or lip movement. After presentation of the verb, participants were asked to respond by typing the verb they believed the actor had conveyed. Results showed that accuracy scores were higher when both visual articulators were present compared to scores with the presence of just one of the modalities (i.e., lip movement). In addition, the enhancement effects of both iconic gestures and lip movements were larger in the 6-band condition compared to the 2-band noise-vocoding condition. From these results, the authors concluded that at a moderate level of noise-vocoding (6-band) (when there are adequate phonological cues in the degraded speech channel), there is an optimal range for maximal multimodal integration where listeners can benefit most from visual information. This finding is consistent with a classic study by Rogers (1978) that examined the effect of visual modalities on the comprehension of speech with signal-to-noise ratios (SNRs) ranging from −8 dB to +7 dB. This study revealed that the effects of visualization of the speaker were greater when the SNR was lower compared to when the SNR was higher. More recently, Holle et al. (2010) utilized functional magnetic resonance imaging (fMRI) to investigate the brain regions in which iconic gestures and speech are integrated by manipulating the signal-to-noise ratio of speech. Results showed greater neural enhancement in the left pSTS/STG when the noise was moderate (SNR −6 dB) compared to when the noise was severe (−14 dB) or good (+2 dB). Thus, both behavioral and neuroimaging studies support the enhancement effects of gestures in noisy speech and in moderate levels of speech degradation.

The gestural enhancement effect on the comprehension of degraded speech has also been observed in studies involving elderly adults (Schubotz et al., 2020) as well as in studies involving non-native listeners (Drijvers and Özyürek, 2020). These studies showed, however, that elderly adults and non-native speakers benefited less from gestures compared to young adults and native listeners, as these groups needed more phonological information to benefit from degraded speech. Elderly adults or non-native listeners could benefit from gestures when speech was less degraded, that is, when more phonological cues were present in the speech signal compared to native adult speakers. Thus, studies with adult participants have shown that gestures can help to disambiguate degraded speech to varying degrees; however, it is unclear to what extent children, who have less experience with speech input than adults, can benefit from gestures when speech is degraded, that is the flexibility of their multimodal integration.

### 1.2 Gesture-speech integration in clear and noisy speech in children

Previous studies have shown that children can process information from gestures and are able to integrate it effectively with clear speech. First of all, children between the ages of 5 and 10 (as well as adults) can obtain gestural information when speech and gestures are presented simultaneously. Additionally, studies have shown that 8- and 10-year-olds are able to detect information conveyed solely in iconic gestures when presented with children’s explanations of Piagetian conservation tasks (Kelly and Church, 1998; Church et al., 2000). Another study revealed the ability of 5- and 6-year-olds to respond to interview questions using information conveyed solely through an interviewer’s iconic gestures (Broaders and Goldin-Meadow, 2010).

Furthermore, research shows that children and adults integrate gestures and speech so that each respective component contributes unique information to the unified interpretation (adults: Cocks et al., 2009; Kelly et al., 2010; children: Kelly, 2001; Sekine et al., 2015). Thus far, two studies have examined children’s ability to effectively integrate iconic gestures and speech. Sekine et al. (2015) examined the ability of both children and adults to integrate speech and iconic gestures in a manner that mutually constrains each other’s meaning. The participants were presented with an iconic gesture, a spoken sentence, or a combination of the two and were instructed to select a photograph that best matched the message communicated. Results showed that 3-year-olds demonstrated increased difficulty integrating information from speech and gesture, but 5-year-olds were able to perform with abilities similar to adults. The researchers concluded that the ability to integrate iconic gestures and speech develops after 3 years of age.

This claim was also supported by a study using electrophysiological measures (Sekine et al., 2020). Sekine et al. examined gesture-speech integration in 6- to 7-year-olds by focusing on the N400 event-related potential (ERP) component, which is modulated by the semantic integration load. The ERPs showed that the amplitude of the N400 was larger in the mismatched gesture-speech condition compared to the matched gesture-speech condition. This result provided neural evidence that children integrate gestures and speech in an online fashion by the age of 6 or 7. Thus, these two lines of study have shown that children are able to collect information from gestures and can integrate it with concurrent speech information.

Although there are no current studies, to our knowledge, that investigate the extent to which iconic gestures assist children with the recognition of speech in adverse conditions, previous research on the recognition of degraded speech alone has revealed that children are able to process degraded speech, albeit not as well as adults (e.g., Eisenberg et al., 2000; Newman and Chatterjee, 2013; Grieco-Calub et al., 2017; Roman et al., 2017). For example, Newman and Chatterjee (2013) assessed the ability of toddlers (27-month-olds) to recognize noise-vocoded speech by comparing their performance with clear speech to their performance with 24-, 8- and 2-band noise-vocoded speech. By measuring the amount of time spent looking at the target picture, they found that toddlers showed equivalent proportions of looking to the target object with clear speech as they did with 24- or 8-band noise-vocoded speech, but they failed to look appropriately with 2-band noise-vocoded speech and showed variable performance with 4-band noise-vocoded speech. These results suggest that even 2-year-olds have developed the ability to interpret vocoded speech; however, children require a long learning period before they are able to recognize spectrally degraded speech as well as adults. Eisenberg et al. (2000) examined the development of the ability to recognize degraded speech by comparing 5- to 7- year-olds, 10- to 12-year-olds, and adults. They presented words or sentences under 4-, 6-, 8-, 16- or 32-noise-band conditions. Results showed that word and sentence recognition scores between adults and older children (10- to 12-year-olds) did not differ statistically. On the other hand, accuracy scores for 5- to 7-year-olds were significantly lower than scores for the other two age groups. Younger children required more spectral resolution (higher than 8-band noise) to perform at the same level as adults and older children. The authors suggested that deficits in younger children are partially due to their inability to fully utilize sensory information and partially due to their incomplete linguistic/cognitive development, including their undeveloped auditory attention, working memory capacity and receptive vocabularies. It is unknown, however, whether children are able to attain the same level of performance as adults when presented with visual cues, such as gestures.

Finally, a few studies have investigated the contribution of visual cues other than gestures, such as visible lip movement, to the comprehension of degraded speech for children. Maidment et al. (2015) explored whether lip movement improves speech identification in typically developing children with normal hearing when the auditory signal is spectrally degraded. They presented sentences that were noise-vocoded with 1–25 frequency bands and compared the task performance (an identification of the color and number presented within spoken sentences) of children aged 4–11 with the performance of adult subjects. They found that the youngest children (aged 4–5) did not benefit from accompanying lip movement in comparison to older children (aged 6–11) and adults who did benefit from visual cues to help recognize degraded speech.

However, it has not been clear to what extent children benefit from hand gestures to comprehend degraded speech and how they compare to adults regarding the different levels of speech degradation. It is possible that while children might be able to integrate iconic gestures with speech in clear speech conditions by ages 6–7 (Sekine et al., 2015, 2020), they might perform less well than adults in doing so when speech is degraded. This could be due to the fact that as children are less proficient at understanding degraded speech, they might also benefit less from gestures than adults, in line with results from non-native and elderly listeners benefit form gestures in noise (e.g., Drijvers and Özyürek, 2017; Schubotz et al., 2020). If this is the case, it shows that even though the multimodal language integration ability in children is there as in adults, it still needs to go through development, especially regarding challenging situations when quality of the input in one channel is not perfect.

### 1.3 Present study

The purpose of the current study was to investigate the enhancement effect of gestures in the comprehension of degraded speech in addition to information gathered from visible speech in children and adults by using noise-vocoded speech. We presented both populations with a word recognition task in two contexts: speech-only or gesture-and-speech combination. By following the previous studies on the effect of gestures on degraded speech in adults (e.g., Drijvers and Özyürek, 2017, 2018, 2020; Schubotz et al., 2020), we made lip movements (visible speech) visible in both contexts. The current study was conducted with 6- and 7-year-olds, as previous research (e.g., Sekine et al., 2020) has shown that children are able to understand and integrate iconic gestures with speech by this age.

Speech quality was manipulated utilizing noise-vocoded speech, which is a speech signal that has been processed to preserve amplitude and temporal information while systematically varying spectral information in the signal (Shannon et al., 1995; Davis et al., 2005). This signal was originally created by Shannon et al. (1995) to simulate perception of speech with a cochlear implant and to investigate the perception of degraded speech in listeners with normal hearing. In this current study, children and adults were given three types of vocoded speech, as well as clear speech with or without gestures with lip visibility in all trials. After the presentation of each stimulus, participants were asked to ‘say’ what they heard into the microphone. We compared the performance of the children with the adults to examine the difference between the two populations and regarding their response to different levels of degradation.

We anticipated that the children would perform more poorly than the adults in the degraded speech-only condition, as shown by Eisenberg et al. (2000), but that it would improve with the input of gestures. Furthermore, we predicted that, with seeing gestures, the children’s performance would be similar to the performance of the adults in the degraded speech-only condition. We also expected the gestural enhancement effect to be greater in adults than in children, due to the fact that children have more difficulty distinguishing phonological cues from degraded speech than adults, thus hindering the benefits of gestures, as shown in the case of elderly adults and non-native speakers (Drijvers and Özyürek, 2020; Schubotz et al., 2020).

Finally, in this study in addition to looking at accuracy we also looked at response times, as measured by the onset of the verbal response. In this aspect, our study differs from Schubotz et al. (2020) that looked at responses to multiple alternatives rather than using a verbal response, which might be a more sensitive measure for response time. Here we expected gestures to facilitate both children and adults’ responses to repeat the speech they heard when gestures were presented compared to instances without gestures. In a gesture, a person’s hand starts moving to prepare the meaningful part of the gesture. This initial phase is called the ‘preparation phase’ and the meaningful phase is called a ‘stroke phase’ (McNeill, 1992). Previous studies (e.g., Sekine and Kita, 2017; Holler et al., 2018) showed that adult listeners responded faster when a speaker produces gestures with speech than when gestures do not occur, because the preparation phase may give a clue about what the speaker will say to the listener. To this end, we calculated response time in both the gesture-speech condition and the speech-only condition in addition to accuracy scores.

## 2 Method

This study was approved by the ethics committee of the Faculty of Arts and the Faculty of Philosophy, Theology and Religious Studies (EAC) at Radboud University in Nijmegen.

### 2.1 Participants

20 adults (mean age = 23, 10 female) and 15 children aged 6–7 years (mean age = 7;02, 8 female. Eight 7-year-olds and seven 6-year-olds) participated in this study. All participants were native, monolingual Dutch speakers. The children were right-handed and had no reported developmental issues. The children’s caregivers did not report any vision or hearing disabilities before testing.

### 2.2 Stimuli

**Figure 1:**
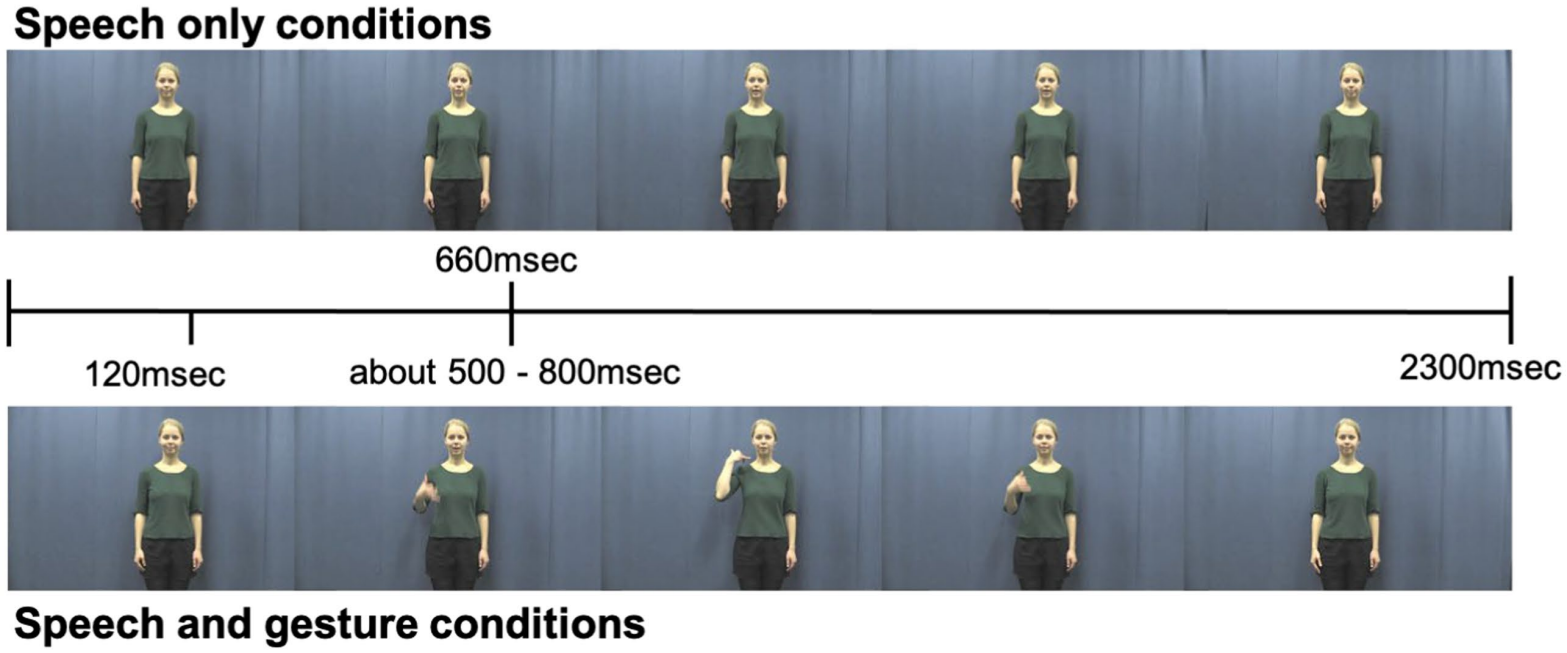
The timeline of video stimuli for speech-only conditions (top panel) and speech + gesture conditions (bottom panel).

170 Dutch action verbs were selected based on the criteria that 80% of 5- and 6-year-old children in the Netherlands are familiar with these verbs (Schaerlaekens et al., 1999; Bacchini et al., 2005; Sekine et al., 2020). In the experiment, 168 of these verbs were used (8 for the practice trial and 160 for experimental trial). Two versions of a video clip (a gesture-and-speech version as well as a speech-only version) were developed for each verb. In the gesture-and-speech version, a native female Dutch speaker produced a Dutch verb with an iconic gesture that depicted the action indicated by the verb (see Figure 1). In the speech-only version, the female produced the same verb without a gesture. The actor was instructed to create the gestures spontaneously and to speak in a child-directed voice. The speech-only video clips were edited so that the onset of speech always began at 660 msec after the onset of the video. Additionally, the gesture-and-speech video clips were edited so that the preparation of the gesture always began 120 msec following video onset. The average onset of stroke phase (the meaningful part of the gesture) was 680 msec (SD = 0.1), and the average onset of speech was 660 msec (SD = 0.1) following video onset (Figure 1). As shown in Figure 1, all video clips were 2,300 msec in length, and the onsets of speech and gestures were very close in timing. These video clips had been used in a previous study involving children in the same age group (Sekine et al., 2020).

The iconic gestures were selected based on pre-test ratings. To ensure that children (a) understood the gestures and (b) could relate them to the relevant verbs, we conducted a pre-test at two elementary schools in the Netherlands. We tested 104 children (M_age_ = 6.74, SD = 0.64) who did not participate in this current study and asked them to rate how much the gesture represented the corresponding verb on a scale from 1 star (not at all) to 5 stars (very much). We selected gestures that had a mean rating was 3 or above (SD ± 1). The further details of the pre-test are described in Sekine et al. (2020).

Three different speech stimuli were utilized for this study: 4-band noise-vocoding speech, 8-band noise-vocoding speech and clear speech. All three stimuli were used with both the adult and child groups. These bands were chosen based on the results of a pilot study in which we discovered that an 8-band noise-vocoded level is a moderate noise level for 6- and 7-year-olds and is the level in which children benefited most from gestures. Further information regarding this study is described in the Supplementary materials. To create noise-vocoded speech, the auditory sound files were separated from the video clips, and the intensity was scaled to 70 dB and de-noised in Praat (Boersma and Weenink, 2015). Noise-vocoded versions were created from the clear audio file for each video by using a custom-made script in Praat. As explained previously, noise-vocoding effectively manipulates varying spectral information while preserving the amplitude and temporal information of the speech signal (Shannon et al., 1995). With this method, the speech signal remains intelligible to a certain extent, depending on the number of vocoding bands, with more bands resulting in a more intelligible speech signal (Davis et al., 2005). By following Drijvers and Özyürek (2017), we filtered each sound file between 50 Hz and 800 Hz and divided the signal into logarithmically spaced frequency bands between 50 and 8,000 Hz. This resulted in cut-off frequencies at 50 Hz, 177.8 Hz, 632.5 Hz, 2249.4 Hz, and 8,000 Hz for 4-band noise-vocoding and 50 Hz, 94.3 Hz, 177.8 Hz, 335.4 Hz, 632.5 Hz, 1192.7 Hz, 2249.4 Hz, 4242.0 Hz, and 8,000 Hz for 8-band noise-vocoding. We used the frequencies to filter white noise in order to obtain 4- and 8- noise bands. The envelope of each band was extracted using half-wave rectification. Then, the amplitude envelope was multiplied with the noise bands, and the bands were recombined to form the distorted signal (Davis et al., 2005). All sound files were recombined with their corresponding video files in Adobe Premiere Pro. Based on the original versions of the video clips and sound files, we created eight different types of audio-visual video clips to include the following experimental conditions: clear speech (CS), clear speech + gesture (CS + G), 4-band noise-vocoded speech (4B), 4-band noise-vocoded speech + gesture (4B + G), 8-band noise-vocoded speech (8B), 8-band noise-vocoded speech + gesture (8B + G), only gesture (OG) (no speech), and only mouth movement (no gesture, no speech). Lip movement was visible in all of the conditions.

Noise-vocoded speech was chosen above other types of noise, such as multi-talker babble noise (e.g., Schubotz et al., 2020), for several reasons. Firstly, noise-vocoded speech ‘enables degradation of the speech signal without adding another *object* to attend, which is the case when there is acoustic competition (i.e., background noise as in multi-talker babble), and which may alter how cognitive resources are distributed during the task’ (Grieco-Calub et al., 2017, p. 3). Additionally, noise-vocoded speech allows researchers to test hypotheses about the effects of spectral degradation on listeners with normal hearing and typically developed auditory and cognitive systems. Lastly, a previous study involving adults examined the enhancement effect of gestures on degraded speech by using noise-vocoded speech (Drijvers and Özyürek, 2017, 2020). We conducted a follow-up study by utilizing the methods and procedures of previous research and adapting them for children.

### 2.3 Procedures

The experiments were conducted individually in a quiet experimental room. Participants were first instructed to sit in front of a computer monitor and put on headphones with a microphone. Then, they were asked to carefully watch and listen to a series of video clips and to verbally repeat the verb they thought the actress in the videos had tried to convey as quickly and as accurately as possible. Lastly, they were provided with an explanation that some of the videos were badly recorded and may contain unclear sounds or not include any sounds at all. Even so, they were instructed to try to say the verb that they thought the actress in the videos had tried to convey.

All video clips were presented on a 15-inch laptop monitor using Presentation software (Neurobehavioral Systems, Inc.). The distance between the monitor and the participant was 60 cm. Each trial began with a fixation-cross for 1,000 msec. Then, the video clip was played for 2,300 msec and followed by a black screen for 4,000 msec (to allow time for a verbal response). The participant’s voice response was recorded from the actor’s speech onset in the video clip until the end of the black screen. After the black screen, a fixation-cross appeared again and was followed by the next video clip.

Each participant completed eight practice trials from each condition in a fixed order. An answer was counted as correct when a participant voiced the correct verb. The response was coded as incorrect when the participant responded with an incorrect verb, an incorrect grammatical category (e.g., responding with a noun rather than a verb), or in instances when the participant did not provide a response. The order of the video stimuli (160 items in total, with 20 items from each condition) was pseudo-randomized and presented in four blocks of 40 trials, with the constraint that a specific condition could not be presented more than twice in a row. Each block consisted of 40 video clips (5 items from each condition). Participants were able to take a self-paced break in between blocks. All participants completed the task in about half an hour. Response time (RT) was measured as the time between the onset of the speech in the video stimulus and the onset of a verbal response.

## 3 Results

### 3.1 Accuracy: comparison between children and adults

**Figure 2:**
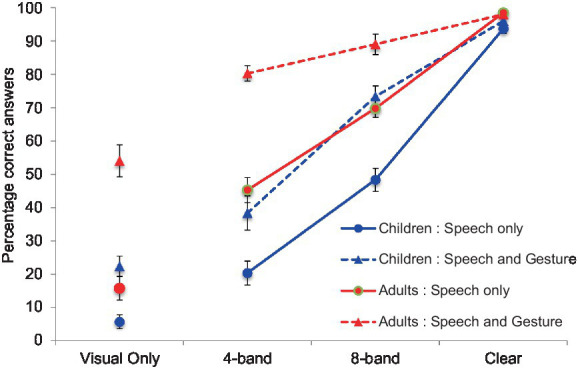
The percentage of correct trials per condition for each age group. The blue lines indicate the children’s results, while the red lines indicate the results from the adult group.

Percentages of correct responses for each group across the eight conditions are presented in Table 1. Each condition included 20 trials. Thus, for example, if a participant responded correctly in five trials (out of 20) in a condition, the correct percentage for the condition was calculated as 25% [e.g., (5/20)*100 = 25]. Figure 2 shows the percentage of correct trials per condition for each age group.

**Table 1:** Mean percentage of correct responses with the standard deviation in parentheses for each group across the eight conditions with the results of paired *t*-tests (*t*-values and effect sides).

Speech quality modality				
Children	Speech only (SO)	Speech and gesture (SG)	*T*-value	Effect size (*r*)
Visual only	5.67 (8.00)	22.33 (11.78)	7.34***	0.89
4ch	20.33 (13.95)	38.33 (20.06)	−4.23***	0.75
8ch	48.33 (13.45)	73.33 (12.49)	−9.83***	0.94
clear	93.67 (5.50)	96 (5.73)	−0.91	0.24
Adults	Speech only (SO)	Speech and gesture (SG)	*T*-value	Effect size (*r*)
Visual only	15.75 (15.84)	54 (21.50)	12.07***	0.94
4ch	45.25 (16.82)	80.25 (10.32)	−13.84***	0.95
8ch	69.75 (12.08)	89 (13.82)	−7.47***	0.86
clear	98.50 (2.86)	98 (3.40)	0.37	0.09

***p < 0.001, **p < 0.01, *p < 0.05.

To investigate potential differences between the children and adults, we performed arcsine transformation for each percentage and then conducted a three-way mixed analysis of variance (ANOVA) with the effect of *modality* [speech-only (SO) vs. speech and gesture (SG)] and *noise-vocoding level* (4-, 8-band noise-vocoding or clear speech) as within-subject factors with age group (children vs. adults) as the between-subject factor for score accuracy. We excluded the two visual-only conditions (gesture and mouth movement and mouth movement only) from the first analysis due to the unbalanced design for ANOVA. Significant main effects were found for *modality*, *F* (1, 33) = 115.22, *p* < 0.001, partial *η*^2^ = 0.77, *noise-vocoding level*, *F* (2, 66) = 409.97, *p* < 0.001, partial *η*^2^ = 0.58, and *age group*, *F* (1, 33) = 46.14, *p* < 0.001, partial *η*^2^ = 0.58. *Post hoc* tests with Bonferroni correction showed that the accuracy scores increased when speech was accompanied by gestures compared to speech presented without gestures. The accuracy score for clear speech was higher than the scores for the two degraded speech conditions, and the adult accuracy score was higher than the children’s accuracy score (all *p* < 0.05).

Additionally, we found a significant interaction between *noise-vocoding level* and *age group*, *F* (2, 66) = 15.50, *p* < 0.001, partial *η*^2^ = 0.32. This effect indicates that noise-vocoding level has a different effect on children than adults. To break down this interaction, contrasts compared each noise-vocoding level across children and adults. These contrasts revealed that the differences in the accuracy score between the 4-band and clear, between the 4-band and 8-band, and between the 8-band and clear speech conditions were significantly larger in children than in adults; *F* (1, 33) = 26.32, *p* < 0.001, partial *η*^2^ = 0.44 for between 4-band and clear speech, *F* (1, 33) = 8.58, *p* < 0.01, partial *η*^2^ = 0.21 for between 4-band and 8-band, and *F* (1, 33) = 6.37, *p* < 0.01, partial *η*^2^ = 0.21 for between 8-band clear speech. These effects showed that although both children’s and adult’s accuracy scores increased with improved speech clarity, this increase was more pronounced for children than for adults.

Furthermore, we also found a significant interaction between *modality* and *noise-vocoding level*, *F* (2, 66) = 22.95, *p* < 0.001, partial *η*^2^ = 0.41, indicating that the effect of gestures on accuracy score differed across noise-vocoding levels. Contrasts compared each noise-vocoding level across speech and gesture (SG) and speech-only (SO) conditions. These contrasts revealed that the accuracy scores in the SG condition were significantly higher compared to the SO condition in 4-band, but there was no significant difference between conditions in clear speech, *F* (1, 33) = 32.39, *p* < 0.001, partial *η*^2^ = 0.50. This was the case for 8-band and clear speech. Although accuracy scores in the SG condition were significantly higher than scores in the SO condition for the 8-band, no significant difference was found for clear speech, *F* (1, 33) = 27.06, *p* < 0.001, partial *η*^2^ = 0.45. These interactions revealed that the effects of gestures on accuracy scores were greater in degraded speech conditions (4- and 8-bands) than in the clear speech condition. This suggests that as speech is degraded, people benefit from gestures. There was no significant interaction between *modality* and *age group*, *F* (1, 33) = 1.02, *p* =. 219.

Finally, we found a significant three-way interaction among *modality*, *noise-vocoding level* and *age group*, *F* (2, 66) = 3.67, *p* < 0.031, partial *η*^2^ = 0.10. This indicates that the interaction between *noise-vocoding level* and *modality* was different in children and adults. Contrasts were used to break down this interaction. These contrasts compared children’s and adults’ accuracy scores at each noise-vocoding level across the two conditions of modality (SG and SO). Contrast revealed a difference between children’s and adults’ accuracy scores when comparing the 4-band condition to clear speech when speech is accompanied with or without gesture, *F* (1, 33) = 6.58, *p* < 0.05, partial *η*^2^ = 0.166. This indicates that for the clear speech condition, accuracy scores were the same across age groups, regardless of whether the speech was accompanied by gestures. However, for the 4-band speech condition, the accuracy score for the speech and gesture (SG) condition was higher for adults (but not for children) than it was for the SO condition. In summary, the three-way interaction indicates that adults benefited from gestures more than children in the 4-band condition. Overall, the three-way mixed ANOVA revealed three findings. First, both children and adults benefited from gestures when speech was degraded. Additionally, adults benefited from gestures more than children when the speech was severely degraded (4-band noise-vocoded speech). Finally, children’s performance in the clear speech condition was similar to the adults’ performance when gestures were presented.

### 3.2 Improvement of children’s performance with gestures

Visual inspection of the data suggested that when gestures were presented, children’s performances in the speech and gesture conditions were similar to the adults’ performances. These results sparked further exploration as to whether children’s performance matches the adults’ (SO condition) level of accuracy when children are given access to gestural information. To explore this possibility, we first compared accuracy scores between adults and children in the SO conditions. We also added a visual-only condition to this analysis so that we could examine comprehension of the target word by only mouth movement (independent of speech) in children and adults. We conducted an independent-samples *t*-test after performing arcsine transformation of each accuracy percentage. Results showed that the accuracy scores in adults were significantly higher than those in children at any noise-vocoded level, including the clear speech condition, *t* (33) = 4.56, *p* < 0.001 for 4-band, *t* (33) = 4.81, *p* < 0.001 for 8-band, *t* (33) = 3.17, *p* < 0.01 for clear speech, and *t* (33) = 2.08, *p* < 0.05 for visual only.

Next, we compared the children’s accuracy scores in the speech and gesture (SG) conditions with the adults’ accuracy scores in the SO conditions with clear speech, 4-band noise-vocoded, 8-band noise-vocoded and visual-only conditions. After arcsine transformation for each accuracy percentage, we conducted independent-samples *t*-tests. As expected, we did not find any significant differences in accuracy scores between children in the SG conditions and adults in the SO conditions at any noise-vocoded levels; *t* (33) = 0.41, *p* = 0.69 for 4-band, *t* (33) = 0.70, *p* = 0.49 for 8-band, *t* (33) = 0.4, *p* = 0.69 for clear speech, and *t* (33) = 1.92, *p* = 0.06 for visual only. This result indicates that when children have access to gestures, their performance approaches the SO performance of the adult group. In addition, because children’s performance improved with the inclusion of gestures, this result confirmed that children can integrate information from gestures and speech.

### 3.3 The gestural enhancement effect in degraded speech between children and adults

**Figure 3:**
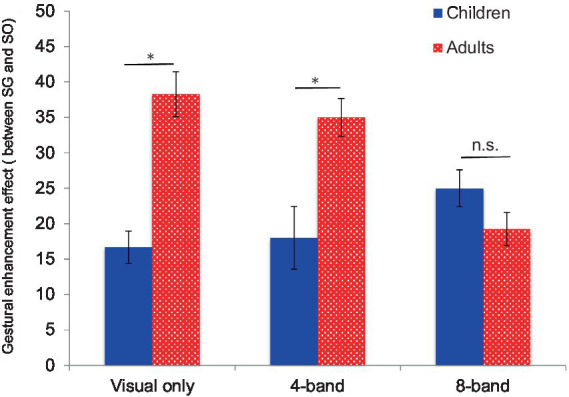
Gestural enhancement effect at each noise-vocoding level for children and adults. Error bars indicate standard errors. *Bonferroni correction (*p* < 0.05).

Despite the analysis in the previous section, we were still unclear on the degree to which adults and children benefit from gestures when speech is degraded. Therefore, we calculated the gestural enhancement effect to measure the degree in which gestures enhance the comprehension of speech. The absolute difference in the percentage of correct trials between the SG and SO conditions was utilized at all speech levels (except for the clear speech condition) to calculate the gestural enhancement effect (Figure 3), in keeping with previous studies of speech recognition performance (e.g., Sumby and Pollack, 1954; Ross et al., 2006). We excluded the clear speech conditions in this analysis because accuracy scores showed celling effects for both SG and SO across both groups.

Following arcsine transformation, we conducted a two-way mixed ANOVA with age group as the between-subject factor (children vs. adults) and the noise-vocoding level (visual-only, 4-band, and 8-band) as the within-subject factor. We found a main effect of age group, *F* (1, 33) = 14.93, *p* < 0.001, partial *η*^2^ = 0.30 and a significant interaction between noise-vocoding level and age group, *F* (2, 66) = 14.47, *p* < 0.001, partial *η*^2^ = 0.31; however, we did not find a main effect for noise-vocoding level, *F* (2, 66) = 1.45, n.s.

For age group, *post hoc* test using the Bonferroni correction (*p* < 0.05) showed that the gestural enhancement effect was generally higher in adults than in children. *Post hoc* tests showed that the enhancement effect was significantly higher for adults compared to children in the 4-band vocoded-noise and the visual-only conditions, but there was no significant difference between adults and children in the 8-band vocoded-noise condition. These results indicate two findings: adults demonstrate increased benefit from gestures compared to children, and both groups demonstrate increased benefit from gestures when the speech signal is moderately (rather than severely) degraded.

### 3.4 Response times: comparison between children and adults

Response times (RTs in ms) were calculated to examine whether gestures facilitate or hinder participants’ word comprehension for each modality and noise-vocoding level. These times were calculated by finding the difference between the speech onset of the video stimulus and the speech onset of participants’ voice responses. Results are shown in Table 2. To this end, we first removed null responses from the dataset and calculated outliers from all RTs. Then, we removed data points that fell above or below two and a half standard deviations from the grand mean. Finally, we averaged the data points for each condition. All of these procedures were conducted for both groups.

**Table 2:** Mean response time (second) and standard deviation in parentheses for each group.

Group	Noise-vocoding level	Speech and gesture	Speech only
Children	4-band	1.543 (0.580)	1.812 (0.397)
	8-band	1.517 (0.533)	1.711 (0.443)
	Clear	1.263 (0.391)	1.495 (0.419)
Adults	4-band	1.360 (0.434)	1.635 (0.421)
	8-band	1.324 (0.452)	1.556 (0.393)
	Clear	1.258 (0.457)	1.463 (0.457)

Next, we conducted a two-way repeated measures ANOVA with *modality* (SG vs. SO) and *noise-vocoding level* (4-band, 8-band, and clear) as the within-subject factor. For children, we found a main effect of modality, *F* (1, 14) = 57.79, *p* < 0.001, *η*^2^ = 0.043, and a main effect of noise-vocoding level, *F* (2, 28) = 39.68, *p* < 0.001, *η*^2^ = 0.069. There was no significant interaction between noise-vocoding level and age group, *F* (2, 28) = 0.43, *p* = 0.65. *Post hoc* tests using the Bonferroni correction (*p* < 0.05) showed that response times in speech and gesture (SG) conditions were significantly faster than in speech-only (SO) conditions. Furthermore, response time in the clear speech condition was significantly faster than the response time for the 4- and 8-band noise-vocoding speech.

For adults, we found a main effect of modality, *F* (1, 19) = 215.23, *p* < 0.001, *η*^2^ = 0.063, and a main effect of *noise-vocoding level*, *F* (2, 38) = 13.27, *p* < 0.001, *η*^2^ = 0.014. There was no significant interaction between *noise-vocoding level* and *age group*, *F* (2, 38) = 1.41, *p* = 0.26. *Post hoc* tests using the Bonferroni correction (*p* < 0.05) showed that response times for the speech and gesture (SG) conditions were significantly faster than the response times in the SO conditions. Additionally, response time in the clear speech condition was significantly faster than the response time for the 4- and 8-band noise-vocoding speech. The response time for the 8-band condition was also significantly faster than the 4-band noise-vocoding speech condition.

## 4 Discussion

The present study investigated to what extent children, compared to adults, benefit from iconic gestures to disambiguate degraded speech by manipulating speech signals and manual modality. The data revealed three overarching findings.

Firstly, both children and adults performed worse with degraded speech compared to clear speech, and children suffered more than adults form degradation as their accuracy of responses to degraded speech was lower than adults. This is consistent with previous research with children (Eisenberg et al., 2000), elderly people (Schubotz et al., 2020), and non-native listeners (Drijvers and Özyürek, 2020), that has shown that degraded speech hinders word comprehension more in such populations than in young adults with native language proficiency.

Secondly, this study revealed that not only adults but also children can benefit from iconic gestures to disambiguate degraded speech. Likewise, this finding is consistent with previous studies with adults revealing that iconic gestures help adult listeners to comprehend information conveyed by speech in noise (Drijvers and Özyürek, 2017; Schubotz et al., 2020; Wilms et al., 2022). This finding is also consistent with previous studies which showed that children can integrate gestures with clear speech (e.g., Kelly and Church, 1998; Church et al., 2000; Kelly, 2001; Broaders and Goldin-Meadow, 2010; Sekine et al., 2015, 2020). However, the findings from the current study provide new evidence to this line of research. That is, children around 7 years of age are capable of gaining information from gestures when speech is degraded and using that information to disambiguate the semantic meaning of the speech.

Interestingly, results revealed that, overall, adults demonstrated greater benefits from iconic gestures at every noise-vocoding level (apart from 8 -band noise-vocoded) compared to children. Furthermore, when gesture was presented in conjunction with speech, children were able to obtain speech comprehension at the level of the adults with SO degraded comprehension. This indicates that, as suggested by Eisenberg et al. (2000), children need more phonological cues to comprehend degraded speech compared to adults. At the same time, children can effectively utilize iconic gestures to enhance the processing of spoken information to reach adult level of degraded speech comprehension. Thus, for children, iconic gestures facilitate disambiguating degraded speech, even though they demonstrate more difficulty processing the spoken channel compared to adults.

However, even though we found the benefit of iconic gestures for children as in adults, our results also show that children need more spectral resolution from speech channels than adults, probably due to children having more difficulties than adults in recognizing the phonological signals from the speech channel. Thus, enhancement from iconic gestures is less in children than in adults.

Thirdly, analysis of response time indicated that iconic gestures help both children and adults in improving the speed of word comprehension, regardless of the quality of speech. When we produce gestures with speech, the hand starts moving (as a preparation phase) to prepare for the meaningful part of the gesture (as a stroke phase) prior to the onset of the corresponding speech (McNeill, 1992; Chui, 2004). Thus, the preparation phase could provide clues to help the participants to roughly predict what meaning will be conveyed by the gesture-and-speech. If this is the case, hand movements of the speaker help facilitate word comprehension for the listeners/observers. In fact, a recent study revealed that when a speaker asked a question with a gesture, he or she received a faster response from listeners compared to speech presented without gesture (Holler et al., 2018). The current study showed that a speaker’s meaningful hand movement can affect word comprehension for the listener and help improve the clarity of communication.

Our findings are in line with Maidment et al. (2015) who showed that children aged 7 years benefit from accompanying visual speech cues. However, their performance is not comparable to the performance of adults (Maidment et al., 2015). This suggests that children need extensive time to develop the ability to disambiguate degraded speech to the same level as adults and that they cannot use multimodal cues as flexibly as adults do. Roman et al. (2017) found that auditory attention and short-term memory capacity were significantly correlated with ability to perceive noise-vocoded words for children aged 5–13 years with normal hearing. In addition to these skills, children need to obtain other language and cognitive skills to utilize gestural information and to predict the words being communicated in order to disambiguate degraded speech. Özer and Göksun (2020) examined adults’ individual difference in gesture-speech integration. They found that people with higher spatial working memory capacity were more efficient in processing gestures whereas people with higher verbal working memory capacity were more sensitive to spoken expressions. This result suggests that children might not develop their ability to integrate gesture and speech uniformly. Thus, as a future task, we recommend further examination of the types of children’s linguistic and cognitive skills (e.g., auditory attention, phonological awareness or working memory capacity) related to the ability to utilize information from gestures to disambiguate degraded speech.

Therefore, overall, this paper shows that while children’s multimodal integration abilities around 7 years of age seems comparable to adults when speech is clear (also shown in our previous research Sekine et al., 2020), this ability might still be developing and not at adult levels in challenging situations when one of the language channels is harder to process. This has implications for our full understanding of the development of a multimodal language system. As a future task, we will examine that the finding from the current study is a robust phenomenon by increasing the number of participants, given that our sample size in this study was relatively small.

Finally, we believe that there are clinical and educational implications of this study. That is, interacting with children as well as adults who are in noisy environments such as classrooms or have hearing difficulties, using gestures might help as gestures help both adults and children to comprehend a speaker’s words, in terms of both accuracy and speed. It would be a future task to find out what type of gestures (e.g., iconic gestures, points, beats etc.) benefits people’s speech comprehension most, and how this benefit interacts with the other cognitive skills of adults and children.

## Data availability statement

The raw data supporting the conclusions of this article will be made available by the authors, without undue reservation.

## Ethics statement

The studies involving humans were approved by the ethics committee of the Faculty of Arts and the Faculty of Philosophy, Theology and Religious Studies (EAC) at Radboud University in Nijmegen. The studies were conducted in accordance with the local legislation and institutional requirements. Written informed consent for participation in this study was provided by the participants’ legal guardians/next of kin. Written informed consent was obtained from the individual(s) for the publication of any potentially identifiable images or data included in this article.

## Author contributions

KS: Data curation, Investigation, Writing – original draft. AÖ: Supervision, Writing – review & editing.
