# Supplementary material for: Children benefit from gestures to understand degraded speech but to a lesser extent than adults
Source: Front Psychol. 2024 Jan 18;14:1305562. doi: 10.3389/fpsyg.2023.1305562 (PMC10832995; doi:10.3389/fpsyg.2023.1305562)
Supplement: Supplementary file 1 [file Data_Sheet_1.pdf]

Supplemental Material

Gestural enhancement effects on children at different noise levels

Before conducting the study reported in the main text, we conducted a pilot study by comparing two groups of 6- and 7-year-olds in order to decide which noise level to use in the current study and to investigate at which noise level children could benefit from gestures the most. The two groups were called Group A and Group B, respectively. Group A consisted of children who eventually participated in the main study. Group B included children who were newly recruited for comparison to the children in Group A. Groups A and B each had 15 children with eight females in each group. The mean age in Group B was 7;02 (Ten 7-year-olds and Five 6-year-olds). Both groups differed in terms of the noise-vocoding levels they were presented with. Group A received the 4- noise-vocoding condition, 8-band noise-vocoding condition and a clear speech condition. Group B received the 8-band noise-vocoding condition, 10-band noise-vocoding condition and a clear speech condition. The creation of the noise-vocoded speech and video stimuli is described in the main text. Cut-off frequencies for the 10-band noise-vocoding were 50 Hz, 83.1 Hz, 138 Hz, 229.2 Hz, 380.7 Hz, 632.5 Hz, 1050.6 Hz, 1745.2 Hz, 2899.1 Hz, 4815.9 Hz, and 8000 Hz.

Correct responses

First, we conducted paired t-tests to compare the mean percentages of correct responses between the speech-only condition and the speech and gesture conditions at each noise-vocoding level for Group B, in the same way we reported our statistical analyses for Group A in the main text. T-values and effect sizes are shown in Table 1 and Figure 1. Group A was also included in Table 1 to note the

differences between the groups. Results revealed that for all groups, the mean percentages of correct responses in the speech and gesture condition were significantly higher than those in the speech-only condition at all noise-vocoding levels (degraded speech or visual-only) except for when the speech was clear and without noise.

Table 1. Mean percentage of correct responses with the standard deviation in parentheses, provided across the eight conditions for each child group. Results of the paired t-tests (t-values and effect sides) are also reported below.

Speech quality	Modality			
**Group A**	**Speech only (SO)**	**Speech and Gesture (SG)**	**T-value**	**effect size (r)**
Visual-only	5.67 (8.00)	22.33 (11.78)	7.34***	0.89
4ch	20.33 (13.95)	38.33 (20.06)	−4.23***	0.75
8ch	48.33 (13.45)	73.33 (12.49)	−9.83***	0.94
clear	93.67 (5.50)	96 (5.73)	−0.91	0.24
**Group B**	**Speech only (SO)**	**Speech and Gesture (SG)**	**T-value**	**effect size (r)**
Visual-only	4.67 (6.40)	18 (10.99)	4.23***	0.75
8-band	45 (15.70)	73.67 (15.52)	−2.76*	0.59
10-band	67.33 (18.41)	76 (18.24)	−7.67***	0.9
Clear	95.33 (5.82)	96.67 (4.50)	−0.65	0.17

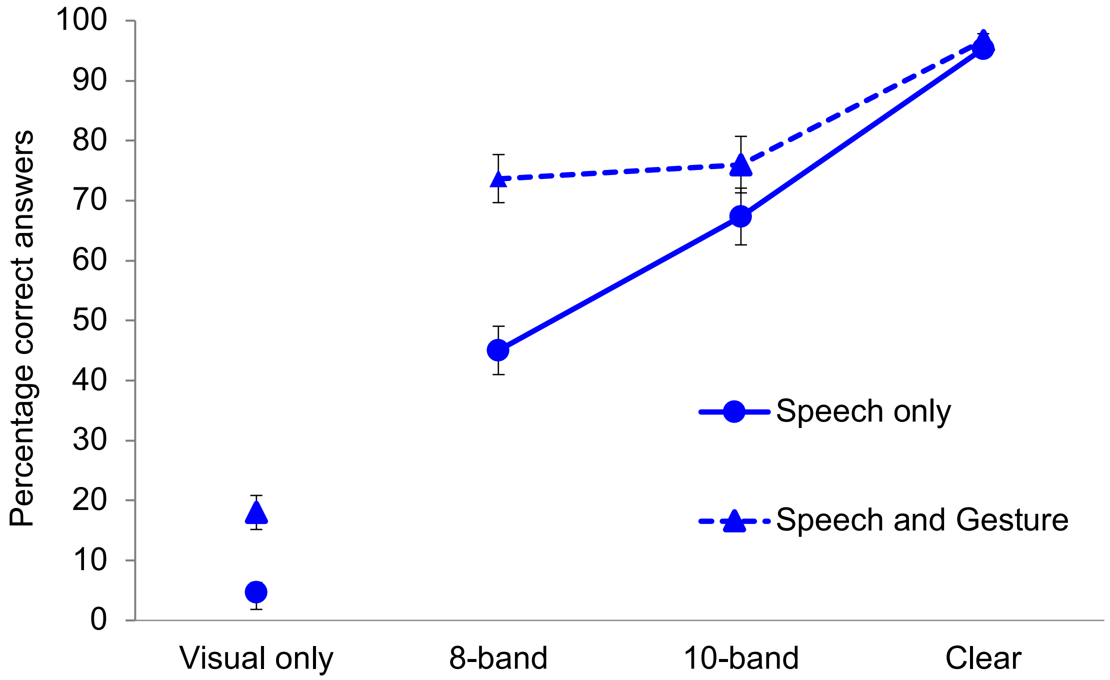

Figure 1. The percentage of correct trials per condition for Group B. The dashed lines indicate results from the speech and gesture (SG) condition, and the solid lines indicate results from the speech-only (SO) condition.

Gestural enhancement effect

In order to investigate the extent to which degraded speech affects children's speech perception, we first examined the differences in gestural enhancement effects at each noise-vocoded level for each child group (Figure 2). We did not directly compare the two groups, as each group was exposed to different noise-vocoded levels. After arcsine transformation for proportional data, we conducted a repeated measure analysis of variance for each child group. The analysis revealed a main effect of *noise-vocoding* for Group A (visual-only vs. 4-band vs. 8-band), F (2, 28) = 3.81, p <.05, partial η^2 =.17, and for Group B (visual-only vs. 8-band vs. 10-band), F (2, 28) = 10.23, p <.001, partial η^2 =.42. Post hoc tests using the Bonferroni correction (p <.05) showed that in Group A, the gestural enhancement effect was

significantly higher in the 8-band condition than in the 4-band and visual-only conditions. The gestural enhancement effect was higher in the 8-band condition than the 10-band and visual-only conditions for Group B. Additionally, statistical analysis revealed that accuracy with the visual-only condition was higher than accuracy for the 10-band noise-vocoding condition. These results indicate that children do not gain much benefit from gestures at 4-band (Group A) and 10-band (Group B) noise-vocoded speech compared to the benefits they gain from gestures with the 8-band noise-vocoded speech. Thus, the 8-band noise-vocoding level appears to be the level in which children benefit most from gestures.

Next, we compared the two child groups to see whether there were differences in the gestural enhancement effect between the 4- (Group A) and 10-band (Group B) noise-vocoding conditions and between the 8-band noise-vocoding conditions across the two child groups. After arcsine transformation, we conducted an independent-samples t-test. Results showed no significant differences (t (28) = 1.49, p =.17) between the 4- and 10-band noise-vocoding conditions, as well as no significant differences (t (28) = 0.91, p =.37) between the 8-band noise-vocoding conditions.

These results indicate that the 8-band noise-vocoded level appears to be the level in which children benefit from gestures the most. The gestural enhancement effect was almost the same for the 4- and 10-band noise-vocoded levels. It can be said that the 8-band noise-vocoded level is the moderate noise level for 6- and 7-year-olds and where children benefit from gestures the most, just as the 6-band noise-vocoded speech is the level in which adults benefit from gestures the most, as shown by Drijven and Özyürek (2017). Thus, we decided to use Group A in the main study.

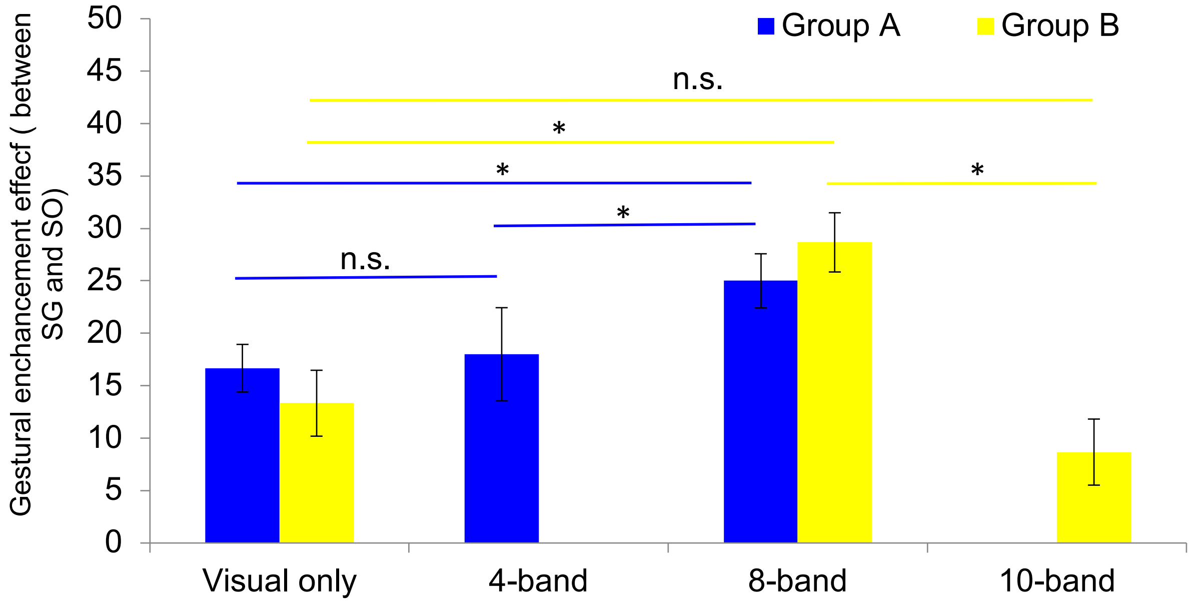

* Bonferroni correction (p < .05)

Figure 2. Gestural enhancement effect at each noise-vocoding level for Groups A and B in the bottom panel. Error bars indicate standard errors.
